# Functional comparison of the HTLV-1 and HTLV-2 antisense viral proteins: implications for pathogenesis

**DOI:** 10.1186/1742-4690-12-S1-O8

**Published:** 2015-08-28

**Authors:** Amanda R Panfil, Nathan Dissinger, Kristina Landes, Patrick L Green

**Affiliations:** 1 Center for Retrovirus Research, The Ohio State University, Columbus, OH 43210, USA; 2 Department of Veterinary Biosciences, The Ohio State University, Columbus, OH 43210, USA; 3 Department of Molecular Virology, Immunology and Medical Genetics, The Ohio State University, Columbus, OH 43210, USA; 4 Comprehensive Cancer Center and Solove Research Institute, The Ohio State University, Columbus, OH 43210, USA

## 

HTLV-1 and HTLV-2 are highly related retroviruses that transform T-lymphocytes in cell culture, but display distinct pathobiology in vivo. HTLV-1 is the causative infectious agent of adult T-cell leukemia/lymphoma (ATL) and a neurodegenerative disease (HAM/TSP), whereas HTLV-2 is nonpathogenic. HTLV-1 encodes a protein on the antisense strand of its proviral genome called HTLV-1 basic leucine zipper (bZIP) factor (HBZ), which inhibits Tax-mediated viral transcription and is required for high proviral load and efficient viral persistence. Studies have shown that HBZ also modulates several cellular pathways that include activating protein-1 (AP-1), NF-κB, and innate immune responses. HTLV-2 also encodes a protein on the antisense genome strand named antisense protein of HTLV-2 (APH-2). Like HBZ, APH-2 also inhibits Tax-mediated viral transcription. However, we show that unlike HBZ, loss of APH-2 results in enhanced viral replication and viral persistence in infected rabbits. This led us to hypothesize that HBZ and APH-2 modulate cellular pathways differently, which translates to the distinct HTLV-1 and HTLV-2 pathobiology. In this study we directly compared APH-2 and HBZ biologic properties and functions on known HBZ-modulated pathways. We provide evidence that APH-2 protein is significantly less stable than HBZ protein (half-life approximately 30m vs. 6.5h). Despite the difference in protein half-life, HTLV-2 does not compensate for this instability by increasing APH-2 mRNA copy number. Additionally, APH-2 and HBZ share similar mRNA stability measurements. We further show that APH-2 inhibits the transforming growth factor β (TGF-β) signaling pathway in contrast to HBZ's enhancement. Like HBZ, APH-2 is able to inhibit the cellular transcription factors p65 (NF-κB) and interferon response factor (IRF)-1. Taken together our results indicate that APH-2 is limited in some functions it shares with HBZ. Further studies should focus on distinct HBZ functions and interacting pathways to find new potential therapeutic targets for HTLV-1 disease.

